# Role of ncRNAs in the Development of Chronic Pain

**DOI:** 10.3390/ncrna11040051

**Published:** 2025-07-03

**Authors:** Mario García-Domínguez

**Affiliations:** 1 Program of Immunology and Immunotherapy, CIMA-Universidad de Navarra, 31008 Pamplona, Spain; mgdom@unav.es; 2 Department of Immunology and Immunotherapy, Clínica Universidad de Navarra, 31008 Pamplona, Spain; 3 Centro de Investigación Biomédica en Red de Cáncer (CIBERONC), 28029 Madrid, Spain

**Keywords:** chronic pain, ncRNA, miRNA, siRNA, lncRNA, circRNA

## Abstract

Chronic pain is a multifactorial and complex condition that significantly affects individuals’ quality of life. The underlying mechanisms of chronic pain involve complex alterations in neural circuits, gene expression, and cellular signaling pathways. Recently, ncRNAs, such as miRNAs, lncRNAs, circRNAs, and siRNAs, have been identified as crucial regulators in the pathophysiology of chronic pain. These ncRNAs modulate gene expression at both the transcriptional and post-transcriptional levels, affecting pain-related pathways like inflammation, neuronal plasticity, and sensory processing. miRNAs have been shown to control genes involved in pain perception and nociceptive signaling, while lncRNAs interact with chromatin remodeling factors and transcription factors to modify pain-related gene expression. CircRNAs act as sponges for miRNAs, thereby influencing pain mechanisms. siRNAs, recognized for their gene-silencing capabilities, also participate in regulating the expression of pain-related genes. This review examines the diverse roles of ncRNAs in chronic pain, emphasizing their potential as biomarkers for pain assessment and as targets for novel therapeutic strategies. A profound understanding of the ncRNA-mediated regulatory networks involved in chronic pain could result in more effective and personalized pain management solutions.

## 1. Introduction

The International Association for the Study of Pain (IASP) describes pain as “an unpleasant sensory and emotional experience often associated with actual or potential tissue damage” [1,2]. This definition highlights the multifaceted nature of pain, encompassing physical and emotional dimensions, and frequently indicating health issues that necessitate medical intervention [3,4,5]. The process of pain perception is initiated by the activation of nociceptors, which are specialized sensory neurons placed in peripheral tissues, including the skin, muscles, and internal organs [6]. These sensory neurons detect harmful stimuli, including mechanical pressure, extreme temperatures, and chemical changes indicative of potential tissue damage [7]. Upon stimulation, nociceptors create electrical signals that travel along afferent nerve fibers to the central nervous system (CNS), where they are processed and interpreted as pain [8]. However, it is important to note that pain involves some pathways within both the peripheral nervous system (PNS) and the CNS, allowing for multiple levels of modulation [9].

The characteristics of pain differ depending on its duration and etiology [10]. Acute pain is commonly brief and has a protective function, indicating immediate tissue injury and inducing behavioral responses to prevent further damages [11]. Conversely, chronic pain persists beyond the usual healing period, usually for years, and can become a debilitating condition affecting both physical and psychological well-being [12]. Chronic pain may arise from conditions including osteoarthritis [13], lower back pain [14], fibromyalgia [15], and cancer [16]. Unlike acute pain, which has a well-defined cause [11], chronic pain can develop into a distinct pathological condition, sometimes in the absence of tissue damage [17].

Chronic pain is particularly prevalent in older adults, as aging is associated with an increased incidence of pain-related conditions, including osteoarthritis, neuropathy and degenerative diseases [18]. Several epidemiological studies in Europe estimate that 38% to 60% of people aged 65 years and older experience chronic pain [19,20]. With advancing age, cumulative health conditions often exacerbate chronic pain, further impairing overall health and quality of life [21]. Several factors contribute to the risk of developing chronic pain, including age, gender, lifestyle and socioeconomic status [12]. Older people are particularly vulnerable due to physiological changes such as reduced tissue elasticity, muscle mass and bone density, all of which increase pain sensitivity and risk of injuries [22]. Gender differences in pain prevalence have also been observed, with women more likely to have chronic pain conditions [23]. In addition, modifiable lifestyle factors including smoking, alcohol consumption, obesity, and sedentary behavior can increase the likelihood of developing or worsening chronic pain [24,25,26].

These factors can influence gene expression and the regulation of pain-related pathways. In recent years, research has increasingly focused on the role of epigenetics in the modulation of chronic pain. Epigenetic modifications, such as DNA methylation [27], histone modifications [28] and non-coding RNAs (ncRNAs) [29], can modify gene expression without altering the underlying DNA sequence. ncRNAs have attracted considerable attention as regulators in the complex mechanisms underlying pain pathways. These molecules play a key role in regulating many biological processes essential for pain perception and response [30]. Unlike traditional protein-coding genes that directly encode proteins, ncRNAs exert their effects through regulatory functions that influence the expression of genes involved in some processes such as inflammation, neuronal plasticity and pain sensitivity [31,32,33].

Specifically, ncRNAs regulate gene expression by interacting with mRNA, chromatin, and other regulatory molecules, thereby influencing the activity of those genes implicated in pain pathways [34]. In the context of inflammation, ncRNAs can either promote or suppress the expression of pro-inflammatory cytokines and other signaling molecules, thus modulating the inflammatory response, which is a crucial contributor to pain [31,35]. Furthermore, it has been demonstrated that ncRNAs play a pivotal role in neuronal plasticity, a key factor in the development of chronic pain [32,36]. By modulating those genes that regulate synaptic strength, receptor sensitivity, and the formation of new neural connections, ncRNAs influence the persistence and exacerbation of pain following injury [37].

In conclusion, this article emphasizes the significant role of ncRNAs in the complex mechanisms that contribute to the onset and development of chronic pain. The following sections will explore the various types of ncRNAs and their roles in pain signaling, neural plasticity, and the regulation of pain-associated genes. A deeper understanding of the molecular interactions and regulatory functions of ncRNAs provides important insights into potential therapeutic approaches for chronic pain.

## 2. MicroRNAs (miRNAs)

MicroRNAs (miRNAs) constitute a subtype of small ncRNA molecules, usually ranging from 21 to 25 nucleotides in length, that act as key regulators of gene expression at the post-transcriptional level across a wide range of organisms, including plants, animals, and viruses [38,39,40]. These ncRNAs regulate gene expression by binding to complementary mRNA sequences, resulting in translational repression or mRNA degradation, ensuring precise and adaptable control of gene activity [41].

**Figure 1:**
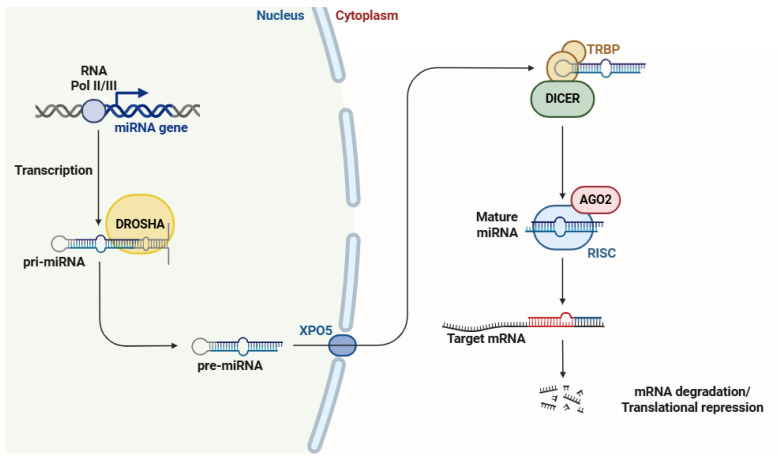
The biosynthesis of miRNAs begins in the nucleus with the transcription of miRNA genes, generating pri-miRNAs. These pri-miRNAs are then processed by the Drosha protein, which enzymatically cleaves them to generate pre-miRNAs. Subsequently, pre-miRNAs are exported to the cytoplasm via the XPO5 protein. Once in the cytoplasm, pre-miRNAs undergo further maturation through the coordinated action of TRBP, Dicer, AGO2, and the RISC protein. This final step yields mature miRNAs, which are responsible for targeting mRNAs. Abbreviations: RNA Pol II/III (RNA polymerases II and III), miRNA (microRNA), pri-miRNA (primary microRNA), pre-miRNA (precursor microRNA), XPO5 (exportin 5), TRBP (transactivation response element RNA-binding protein), AGO2 (argonaute 2), RISC (RNA-induced silencing complex), and mRNA (messenger RNA). Figure adapted from Ref. [51].

The biogenesis of miRNAs (Figure 1) is a multistep process that starts in the cell nucleus, where they are transcribed as primary miRNAs (pri-miRNAs) by RNA polymerase II, or in some cases, RNA polymerase III [42,43]. pri-miRNAs are further recognized and processed by the Drosha–DGCR8 microprocessor complex, which cleaves them into precursor miRNAs (pre-miRNAs) that are transported to the cytoplasm via exportin-5 (XPO5) in a RanGTP-dependent manner [44,45]. In the cytoplasm, pre-miRNAs are further cleaved by the endoribonuclease Dicer, resulting in the formation of mature miRNA duplexes [46]. From miRNA duplexes, only one strand (guide strand) is selectively incorporated into the RNA-induced silencing complex (RISC), while the passenger strand is commonly degraded [47]. Once integrated into the RISC, the miRNA directs the complex to specific mRNAs based on sequence complementarity, with binding primarily occurring in the 3′ untranslated region (3′ UTR) of the target gene [48], leading to either mRNA cleavage and degradation [49] or translation repression [50].

This regulatory mechanism allows miRNAs to modulate gene networks with exceptional specificity and versatility, impacting a wide array of biological processes, including cell proliferation and differentiation [52], apoptosis [53], stress response [54], and immune function [55]. Furthermore, miRNAs are essential in intercellular communication by being released into extracellular fluids like blood, saliva, and urine. They are either encapsulated within exosomes and microvesicles [56,57,58] or associated with RNA-binding proteins such as argonaute (AGO), thereby functioning as systemic signaling molecules [59]. Given their crucial role in maintaining cellular homeostasis, disruptions in miRNA function are associated with numerous diseases, such as cancer [60], neurodegenerative disorders [61], cardiovascular diseases [62], and autoimmune conditions [63], positioning them as promising candidates for therapeutic intervention and biomarker discovery [64].

Advancements in bioinformatics and high-throughput sequencing technologies, like microarrays [65], RNA sequencing [66], and cross-linking immunoprecipitation (CLIP) assays [67], have enabled the identification of miRNA targets and expression patterns under several biological conditions, providing a more profound understanding of their biological functions [68]. As research continues to elucidate the complexities of miRNA networks, it has become increasingly clear that these small molecules exert remarkable control over gene expression, functioning as master regulators with significant implications for precision medicine, regenerative therapies, and disease intervention strategies [69].

Numerous studies have shown that certain miRNAs are upregulated in response to inflammatory mediators, playing a role in the enhancement of pain signaling by regulating the expression of cytokines, receptors, and ion channels involved in pain transmission [70,71]. In contrast, other miRNAs have been shown to attenuate excessive pain responses and promote various analgesic pathways [72,73]. Furthermore, miRNAs are implicated in the modulation of synaptic plasticity, a key process in the transition from acute to chronic pain, by influencing the expression of biomolecules linked to long-term potentiation (LTP) and long-term depression (LTD) in pain-processing areas including the spinal cord and brain [74]. Finally, recent studies have highlighted the potential of miRNAs as diagnostic biomarkers for chronic pain, as their expression profiles reflect the underlying pathological changes in pain pathways [75].

Table 1 outlines the altered expression of miRNAs in various preclinical chronic pain conditions, including inflammatory, neuropathic, and cancer-related pain. These miRNAs could serve as potential biomarkers or therapeutic targets.

## 3. Small Interfering RNAs (siRNAs)

**Figure 2:**
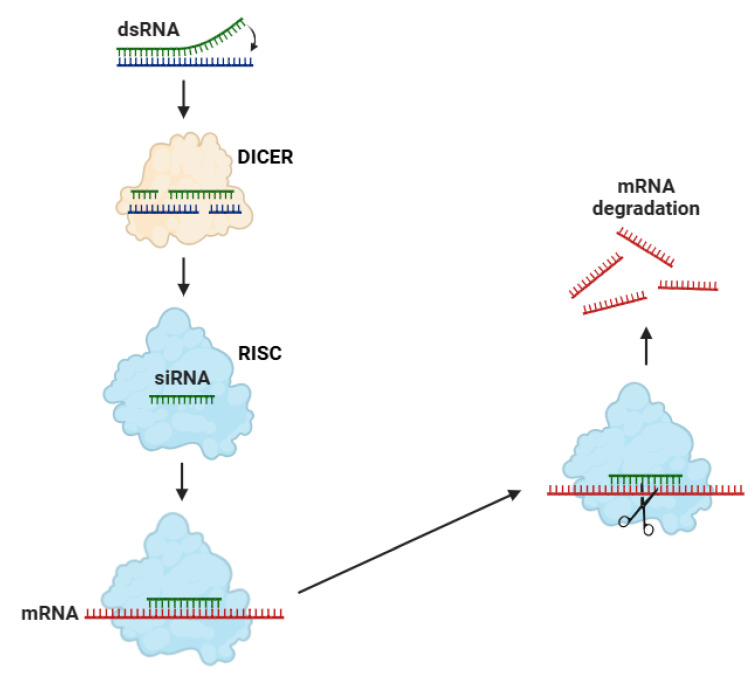
The biosynthesis of siRNAs begins with the formation of dsRNA, which is recognized and cleaved by the enzyme Dicer into small interfering siRNAs. One strand of the siRNA is then incorporated into the RISC, guiding it to a complementary mRNA. RISC binds to the target mRNA and cleaves it at a specific site, contributing to its degradation and preventing protein biosynthesis. Abbreviations: dsRNA (double-strand RNA), RISC (RNA-induced silencing complex), and mRNA (messenger RNA). Figure adapted from Ref. [153].

Small interfering RNAs (siRNAs) are fundamental elements of the RNA interference (RNAi) pathway, a highly conserved regulatory mechanism that facilitates post-transcriptional gene silencing [149]. The biosynthesis of siRNAs, characterized as double-stranded RNA (dsRNA) molecules, initiates in the cytoplasm with the cleavage of endogenous or exogenous dsRNA substrates by the enzyme Dicer (Figure 2) [150]. Dicer, in combination with some cofactors (such as TRBP) in mammals, recognizes and cleaves the dsRNA into siRNA duplexes with characteristic two-nucleotide 3′ overhangs [151]. Subsequently, the siRNA duplexes are loaded into the RISC, where they undergo strand selection, a process mediated by the helicase activity of AGO proteins [152].

The strand with the thermodynamically less stable 5′ end is preferentially retained as the guide strand, while the passenger strand is degraded [154,155]. The guide strand, incorporated into the mature RISC, facilitates sequence-specific gene silencing by recognizing and binding to complementary target mRNAs [155]. This binding enables the AGO-mediated endonucleolytic cleavage of the target transcript, leading to its degradation and preventing translation [156]. In certain organisms, amplification of the RNAi response occurs through RNA-dependent RNA polymerases (RdRPs), which generate secondary siRNAs from cleaved target mRNAs, thereby enhancing gene silencing [157,158].

A key feature of siRNAs is their antiviral function, where they specifically recognize and degrade viral RNA, thereby preventing viral replication [159]. Their ability to disrupt viral genomic material limits the progression of infection and enhances the host’s capacity to initiate an immune response [160]. In addition to their antiviral properties, siRNAs are crucial for maintaining genomic stability. siRNAs are crucial in silencing transposable elements, which are mobile genetic components capable of moving within the genome, potentially causing mutations and genomic instability [161], or contributing to the development of diseases such as cancer [149], neurodegenerative disorders [162], and genetic syndromes [163]. Conversely, siRNAs are involved in modulating gene expression, influencing the activation or repression of specific genes in response to various internal and external stimuli [164]. This function extends to cellular differentiation, where siRNAs contribute to the determination of cell fate during development, ensuring the proper identity and function of cells within tissues [165]. Moreover, siRNAs mediate many cellular responses to environmental stressors, including alterations in temperature, nutrient availability, and exposure to toxins [164].

Recent studies have highlighted the potential of siRNAs in pain management by selectively modulating the expression of genes involved in pain pathways. Through the targeted silencing of key molecules such as inflammatory cytokines [166], ion channels [167], and neurotransmitters [168], siRNAs offer a promising approach to developing effective and personalized therapies for chronic pain. Table 2 provides an overview of the siRNAs used for treating inflammatory, neuropathic, and cancer-related pain.

## 4. Long Non-Coding RNAs (lncRNAs)

Long non-coding RNAs (lncRNAs) represent a complex class of non-protein-coding transcripts that exceed 200 nucleotides in length [201]. lncRNAs have emerged as key regulators of gene expression at multiple levels, including chromatin remodeling [202], transcriptional and post-transcriptional modulation [203,204] across many organisms. Unlike miRNAs and siRNAs, which mainly function through base-pairing interactions with target mRNAs [41,154], lncRNAs regulate gene expression via a broad spectrum of mechanisms, acting as molecular scaffolds, decoys, sponges, and guides [205]. By facilitating the recruitment of specific protein complexes to genomic loci, they regulate gene expression in a context-dependent manner. However, developments in technology have shown that numerous lncRNAs contain small open reading frames (sORFs) capable of encoding micropeptides. These micropeptides mediate key biological functions, including the regulation of homeostasis, inflammation, immune responses, metabolism, and tumor progression [206].

**Figure 3:**
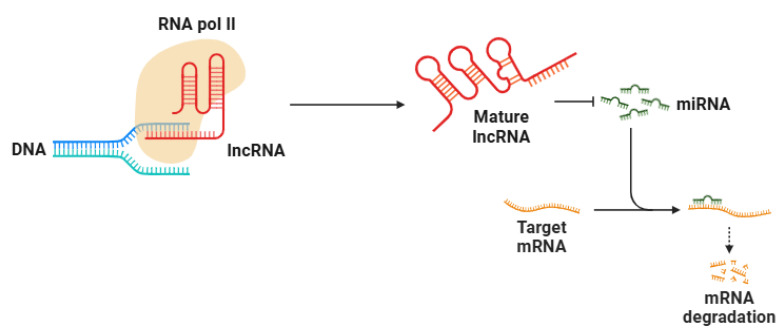
lncRNAs are mainly transcribed by RNA polymerase II from non-coding genomic regions. Their biosynthesis closely resembles mRNA processing, encompassing transcription initiation, elongation, splicing, 5′ capping, and 3′ polyadenylation. lncRNAs act as molecular sponges for miRNAs, inhibiting miRNA-mediated repression of target genes. Abbreviations: RNA pol II (RNA polymerase II), lncRNA (long non-coding RNA), miRNA (microRNA), and mRNA (messenger RNA). Figure created with BioRender 23.

The biogenesis of lncRNAs (Figure 3) shares similarities with that of protein-coding mRNAs. They are transcribed by RNA polymerase II and undergo several post-transcriptional modifications, including the addition of a 5′ cap, polyadenylation at the 3′ end, and splicing [207]. Despite these similarities, lncRNAs lack protein-coding potential due to the absence of long open reading frames (ORFs) and ribosomal translation initiation signals [208]. While some lncRNAs are polyadenylated, others remain non-polyadenylated, adding another layer of complexity to their function [209,210]. The expression of lncRNAs is often highly cell-type-specific and tightly regulated, with distinct spatial localization patterns that contribute to their functional diversity [211].

Depending on their localization, lncRNAs can exert their effects either within the nucleus or the cytoplasm. Nuclear lncRNAs play crucial roles in chromatin architecture and epigenetic modifications, often interacting with chromatin-modifying complexes such as Polycomb Repressive Complex 2 (PRC2) to induce gene silencing through histone modifications [212,213]. Furthermore, lncRNAs can recruit transcription factors to specific genomic loci, regulating transcription in a gene-specific fashion [214]. Conversely, in the cytoplasm, lncRNAs affect mRNA stability, translation, and post-transcriptional regulation by interacting with RNA-binding proteins, miRNAs, and ribosomes [215]. One intriguing mechanism is their ability to function as competing endogenous RNAs (ceRNAs), wherein lncRNAs act as molecular sponges for miRNAs, thereby preventing miRNA-mediated repression of target genes [216]. This ceRNA network adds another regulatory layer to gene expression and has significant implications in various biological processes [217].

Functionally, lncRNAs are deeply embedded in numerous physiological processes, including cell differentiation [218], proliferation [219], apoptosis [220], immune responses [221], and genomic imprinting [222]. Furthermore, recent evidence indicates that lncRNAs can be involved in intercellular communication by being selectively incorporated into extracellular vesicles, such as exosomes [223]. Once secreted, these lncRNA-containing vesicles can be taken up by recipient cells, where they influence gene expression and cellular behavior, further highlighting their role in systemic regulation [223].

Given their regulatory roles, dysregulation of lncRNAs has been strongly associated with various diseases, including cancer [224], neurodegenerative disorders [225], cardiovascular diseases [226], and metabolic syndromes [227]. In cancer, dysregulated lncRNA expression can play a role in tumor initiation, progression, metastasis, and resistance to therapy by modulating oncogenic and tumor-suppressor pathways [224]. Some lncRNAs function as oncogenes, promoting tumor growth and invasion, while others act as tumor suppressors, inhibiting malignancy [228]. In neurodegenerative disorders, lncRNAs have been implicated in neuroinflammation, synaptic dysfunction, and protein aggregation, suggesting their potential as therapeutic targets [225]. On the other hand, in cardiovascular diseases, lncRNAs participate in cardiac hypertrophy and atherosclerosis, further emphasizing their broad biological significance [226]. Techniques such as RNA sequencing (RNA-seq) [229], chromatin isolation by RNA purification (ChIRP) [230], and CLIP [231] have provided valuable insights into lncRNA functions and their molecular interactions. These methodologies have provided a substantial toolkit for dissecting the complex regulatory networks mediated by lncRNAs. By mapping the interactions between lncRNAs and the DNA-RNA-protein complex, researchers can construct molecular interaction networks that reveal the roles of lncRNAs in gene regulation.

In the field of chronic pain research, increasing evidence suggests that lncRNAs play a significant role in modulating pain by regulating the expression of pro-nociceptive and anti-nociceptive genes, influencing neuroinflammatory pathways, and affecting synaptic plasticity in critical pain-processing regions such as the spinal cord and brain [232]. Some lncRNAs have been shown to contribute to pain sensitization through interactions with inflammatory cytokines, ion channels, and intracellular signaling pathways involved in pain transmission [233].

Finally, lncRNAs have emerged as valuable diagnostic biomarkers for chronic pain conditions, as their expression patterns reflect underlying pathological changes in pain-processing circuits. The detection of several lncRNAs in biofluids including blood [234], cerebrospinal fluid [235], and saliva [236] makes them promising candidates for non-invasive pain diagnostics. Identifying distinct lncRNA signatures associated with various pain conditions could support the development of precision medicine approaches, enabling personalized therapies tailored to individual patients [237]. Table 3 summarizes the alterations in the expression of lncRNAs observed across various preclinical models of chronic pain, including those related to inflammation, nerve injury, and cancer. These changes in lncRNA expression are significant as they could serve as biomarkers for the diagnosis of chronic pain conditions or as targets for the development of novel therapeutic approaches aimed at pain relief. Targeting these lncRNAs could facilitate the development of effective treatments for individuals afflicted with different types of chronic pain.

## 5. Circular RNAs (circRNAs)

Circular RNAs (circRNAs) have attracted significant attention owing to their distinctive and remarkable structural characteristics, which differentiate them from conventional linear RNAs. Unlike linear RNAs that possess a 5′ cap and a 3′ poly(A) tail [252], circRNAs are distinguished by their covalently closed loop structures, which render them more stable and resistant to exonuclease-mediated degradation [253]. This stability contributes to the prolonged presence of circRNAs in various bodily fluids, positioning them in a wide range of physiological and pathological processes [254].

**Figure 4:**
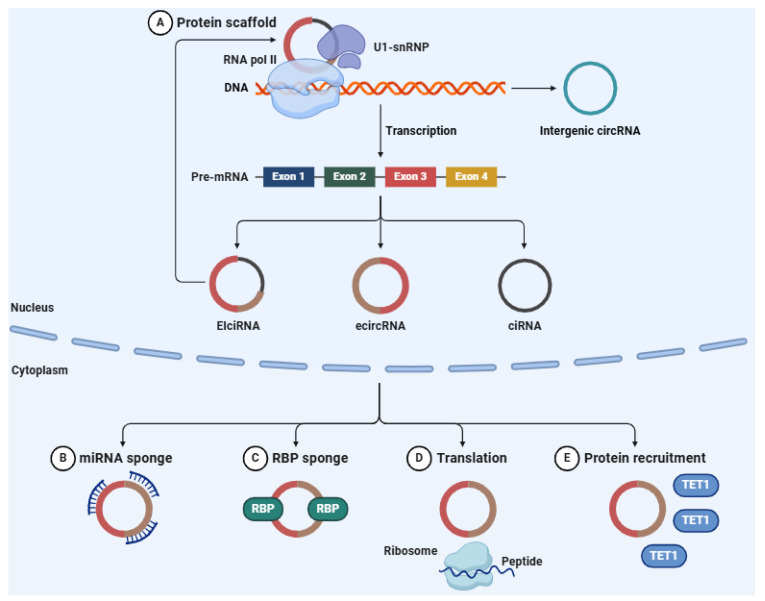
circRNAs are generated via the back-splicing of precursor mRNAs, resulting in RNA molecules that are covalently closed. These circRNAs participate in the regulation of gene expression by functioning as molecular sponges for miRNAs. Through their interaction with miRNAs, circRNAs inhibit their ability to target mRNAs, thereby modulating gene expression. In a similar fashion, circRNAs can sequester RBPs, influencing several processes such as translation, protein recruitment, and RNA processing. Abbreviations: U1-snRNP (U1 small nuclear ribonucleoprotein), RNA pol II (RNA polymerase II), DNA (deoxyribonucleic acid), circRNA (circular RNA), pre-mRNA precursor messenger RNA), EIciRNA (exon-intron circRNA), ecircRNA (exonic circRNA), ciRNA (circular intronic RNA), miRNA (microRNA), RBP (RNA-binding protein), TET1 (ten-eleven translocation methylcytosine dioxygenase 1). Figure adapted from an original illustration created with BioRender 23 (Authorship: Martina Maritan).

CircRNAs arise from pre-mRNAs via a non-canonical splicing mechanism known as back-splicing (Figure 4). In this process, a downstream splice donor site is connected to an upstream splice acceptor site, leading to the formation of a circRNA [255]. This back-splicing event contrasts with the typical linear splicing processes involved in the production of conventional mRNA molecules [256]. The different structural forms of circRNAs, including exonic circRNAs (ecircRNAs), circular intronic RNAs (ciRNAs), and exon-intron circRNAs (EIciRNAs), further support their roles in gene expression regulation and cellular functions [257,258,259]. The biogenesis of circRNAs is regulated by a host of factors, such as RNA-binding proteins (RBPs), spliceosome components, and cis-regulatory elements [260,261]. These factors help facilitate the back-splicing process, ensuring that circRNAs are produced in a controlled and context-specific manner. Key RBPs like MBL (mannose-binding lectin) and FUS (fused in sarcoma) are crucial for stabilizing circRNA formation by either aiding intronic base-pairing or recruiting the splicing machinery [262,263]. Furthermore, the complementary sequences within intronic regions play a critical role in determining the type and diversity of circRNAs that are expressed across different tissues and developmental stages [264].

CircRNAs have been identified as important regulators of gene expression, particularly through their interactions with different miRNAs. One of the most documented roles of circRNAs is their capacity to function as molecular sponges for miRNAs [265]. By sequestering miRNAs, circRNAs prevent them from interacting with their target mRNAs, thereby modulating gene expression at the post-transcriptional level [266]. In addition to acting as miRNA sponges, circRNAs have also been implicated in modulating transcription, adjusting alternative splicing events, and even regulating translation [267,268,269]. Certain circRNAs have been shown to harbor internal ribosome entry sites (IRESs) or post-transcriptional modifications, such as N6-methyladenosine (m6A), which enable them to serve as templates for translation into functional peptides [270,271]. These functions underscore the versatility of circRNAs in cellular regulation and their potential involvement in many cellular processes, from proliferation to apoptosis [272]. The dysregulation of circRNAs has been implicated in a diverse array of pathological conditions, including cancer [273], neurodegenerative diseases [274], and cardiovascular disorders [275].

The role of circRNAs in sensory processing represents an energizing area of research, with emerging evidence suggesting that these ncRNAs may contribute significantly to the pathophysiology of pain. Within the context of chronic pain, specific circRNAs have been shown to be upregulated in pain-associated tissues, including the DRG (dorsal root ganglion) and spinal cord [276,277]. These circRNAs interact with miRNAs, thereby influencing the expression of genes that regulate pain signaling pathways. For instance, circRNAs regulate the expression of ion channels, like ASICs (acid-sensing ion channels), that are essential for the transmission of pain signals [278]. Moreover, several circRNAs have been shown to modulate the activity of pain-related transcription factors, such as AP-1 (activator protein 1) and NF-κB (nuclear factor kappa-light-chain-enhancer of activated B cells), as well as signaling molecules including cytokines and neurotrophins, which are pivotal in the sensitization of nociceptors [279,280,281,282]. Through their ability to influence the expression of those genes involved in inflammation, neuronal excitability, and synaptic plasticity, circRNAs contribute to the complex network that regulates many chronic pain responses [283,284,285]. Especially, circRNAs that modulate numerous inflammatory responses have been shown to influence the onset and persistence of inflammatory pain. The dysregulation of circRNAs may contribute to the maintenance of chronic pain states by altering the balance between pro-inflammatory and anti-inflammatory signaling [286].

Finally, given their role in regulating gene expression and several cellular processes, circRNAs hold immense potential as both diagnostic biomarkers and therapeutic targets for diseases associated with chronic pain [287]. The implications of circRNAs in pain represent a promising frontier in both basic research and clinical applications. By understanding how circRNAs contribute to pain modulation and chronic pain conditions, researchers may develop novel therapeutic strategies aimed at alleviating pain through the manipulation of circRNA function [288,289]. In this way, circRNAs may emerge as key players in the next generation of pain management therapies, potentially offering new hope for patients suffering from chronic pain disorders.

Table 4 presents a comprehensive overview of circRNA expression alterations identified in various preclinical models of chronic pain, including those linked to inflammation, neuropathies, and cancer. These alterations are highly relevant, as they could be used as biomarkers for the diagnosis of chronic pain or as targets for the development of novel therapeutic approaches in pain management.

## 6. Conclusions

The role of ncRNAs in the development of chronic pain is increasingly recognized as a key area of research with significant therapeutic potential (Table 5). ncRNAs, including miRNAs, siRNAs, lncRNAs, and circRNAs, have emerged as pivotal regulators of gene expression, impacting numerous cellular processes linked to pain perception and chronic pain pathophysiology. These biomolecules regulate the expression of pain-related genes, influence neuroinflammation, alter ion channel function, and affect the plasticity of neural circuits involved in pain processing. Dysregulation of ncRNA has been associated with some chronic pain conditions, underscoring their role as potential biomarkers for diagnosis and targets for treatment.

A growing body of evidence supports the differential expression of numerous classes of ncRNAs in both animal models and human patients with chronic pain conditions, including neuropathic, inflammatory, and cancer-related pain. These results emphasize the prospective utility of ncRNAs as minimally invasive diagnostic biomarkers, detectable in biofluids including blood and cerebrospinal fluid, offering new promising possibilities for early detection, pain subtyping, and monitoring of treatment responses [307]. Additionally, recent research highlights that miRNAs function as suppressors of mRNA translation, leading to reduced protein levels of pain modulators, while lncRNAs and circRNAs act as molecular sponges, sequestering miRNAs and influencing their regulatory activities. siRNAs also play a crucial role in this regulatory network by promoting the degradation of specific mRNA. The complex interactions between these ncRNAs and their target molecules suggest a sophisticated regulatory network that contributes to both the persistence and intensification of chronic pain states. Understanding these complex interactions opens new avenues for the development of ncRNA-based therapeutics, such as miRNA mimics [308], antagomirs [309], lncRNA modulators [310], and siRNA-based treatments [311], aiming to restore normal gene expression patterns and alleviate pain.

However, despite these advances, significant barriers hinder the clinical translation of ncRNA-based diagnostics and therapeutics. These include challenges related to the stability, specificity, and immunogenicity of RNA-targeting agents, efficient delivery across biological barriers (particularly the blood–brain barrier), and the risk of off-target effects due to the pleiotropic and context-dependent nature of ncRNA function [312]. Interindividual variability in ncRNA expression profiles, differences across pain etiologies, and the current lack of standardized detection and quantification methods further complicate clinical implementation [313]. To overcome these limitations, future research should prioritize the development of targeted delivery systems (e.g., nanoparticle-based platforms), refine computational tools for predicting ncRNA-target interactions, and conduct clinical studies to validate candidate ncRNAs across diverse patient populations [314,315,316]. In parallel, regulatory frameworks must adapt to the unique characteristics of RNA-based therapeutics. Through the resolution of these challenges, the field can advance toward the clinical integration of ncRNA-based strategies, ultimately contributing to more precise, personalized, and effective approaches to chronic pain management.

In conclusion, ncRNAs show significant potential for elucidating the complex mechanisms underlying chronic pain and developing innovative strategies. Continued exploration of ncRNA biology will be essential to overcoming current limitations and harnessing their full therapeutic potential in the management of chronic pain.

## Figures and Tables

**Table 1:** List of dysregulated miRNAs across chronic pain disorders. Abbreviations: SNL (spinal nerve ligation), CCI (chronic constriction injury), CIPN (chemotherapy-induced neuropathic pain), CFA (complete Freund’s adjuvant), SNI (spared nerve injury), CIVP (chronic inflammatory visceral pain), DPN (diabetic peripheral neuropathy), SCI (spinal cord injury), BCP (bone cancer pain), CRC (colorectal cancer), bCCI (bilateral chronic constriction injury), CCI-IoN (chronic constriction injury of the infraorbital nerve), CCD (chronic compression of the DRG), L5-VRT (L5 ventral root transection), AIA (adjuvant-induced arthritis), and BTZ (bortezomib).

miRNA	Pain Condition	Pain Model	Effects on Pain	References
miR-7	Neuropathic pain	SNL	Anti-hyperalgesic (miR-7a)	[76]
miR-15	Neuropathic pain	CCI	Anti-hyperalgesic (miR-15a)	[77]
		CIPN (Oxaliplatin)	Hyperalgesic (miR-15b)	[78]
miR-16	Inflammatory pain	CFA	Anti-hyperalgesic	[79]
	Neuropathic pain	CCI		[80]
miR-20	Neuropathic pain	CCI	Anti-hyperalgesic (miR-20b-5p)	[81]
miR-21	Neuropathic pain	SNI	Hyperalgesic (miR-21-5p)	[82,83]
miR-26	Neuropathic pain	CCI	Anti-hyperalgesic (miR-26a-5p)	[84]
	Inflammatory pain	CFA		[85]
miR-29	Inflammatory pain	CFA	Hyperalgesic (miR-29a)	[86]
	Neuropathic pain	SNI	Hyperalgesic (miR-29c)	[87]
	Inflammatory pain	LPS	Anti-hyperalgesic (miR-30a-5p)	[88]
miR-30	Neuropathic pain	CCI	Anti-hyperalgesic (miR-30b-5p)	[89]
		SNI	Anti-hyperalgesic (miR-30c-5p)	[90]
miR-34	Neuropathic pain	CCI	Anti-hyperalgesic (miR-34a)	[91]
			Anti-hyperalgesic (miR-34c)	[92]
	Inflammatory pain	CFA	Anti-hyperalgesic (miR-34a)	[93]
		CIVP		[94]
miR-101	Neuropathic pain	CCI	Hyperalgesic	[95]
			Anti-hyperalgesic	[96]
miR-103	Neuropathic pain	SNL	Anti-hyperalgesic	[97]
miR-107	Inflammatory pain	CFA	Hyperalgesic	[98]
miR-124	Neuropathic pain	CCI	Anti-hyperalgesic (miR-124-3p)	[99]
		SNL		[100]
	Inflammatory pain	CFA		[101]
miR-125	Inflammatory pain	CFA	Anti-hyperalgesic (miR-125a-3p)	[102]
	Neuropathic pain	DPN	Anti-hyperalgesic (miR-125a-5p)	[103]
		CCI	Anti-hyperalgesic (miR-125b-5p)	[104]
miR-128	Neuropathic pain	CCI	Anti-hyperalgesic (miR-128-3p)	[105]
		SCI		[106]
miR-130	Neuropathic pain	SCI	Hyperalgesic (miR-130a-3p)	[107]
			Hyperalgesic (miR-130a-5p)	[108]
miR-132	Neuropathic pain	SNI	Hyperalgesic (miR-132-3p)	[109]
miR-134	Neuropathic pain	CCI	Anti-hyperalgesic (miR-134-5p)	[110]
	Inflammatory pain	CFA	Hyperalgesic	[111]
miR-135	Neuropathic pain	CCI	Hyperalgesic (miR-135a-5p)	[112,113]
	Cancer pain	BCP	Anti-hyperalgesic (miR-135-5p)	[114]
miR-142	Neuropathic pain	SNL	Anti-hyperalgesic (miR-142-3p)	[115]
		CCI	Anti-hyperalgesic (miR-142-5p)	[116]
miR-146	Neuropathic pain	SNL	Anti-hyperalgesic (miR-146-3p)	[117]
		CCI		[118]
	Cancer pain	CRC	Anti-hyperalgesic (miR-146a)	[119]
miR-155	Neuropathic pain	bCCI	Hyperalgesic	[120]
		DPN		[121]
miR-181	Neuropathic pain	SNL	Anti-hyperalgesic (miR-181b)	[122]
		CCI	Anti-hyperalgesic (miR-181c-5p)	[123]
miR-183	Neuropathic pain	CCI	Anti-hyperalgesic	[124,125]
miR-190	Neuropathic pain	DPN	Anti-hyperalgesic (miR-190a-5p)	[126]
miR-195	Neuropathic pain	SNL	Hyperalgesic	[127]
		CCI-IoN		[128]
miR-199	Neuropathic pain	CCD	Anti-hyperalgesic	[129]
	Cancer pain	BCP		[130]
miR-203	Neuropathic pain	bCCI	Hyperalgesic	[131]
miR-212	Neuropathic pain	CCI	Anti-hyperalgesic (miR-212-3p)	[132]
		SCI		[133]
miR-219	Inflammatory pain	CFA	Anti-hyperalgesic	[134]
miR-223	Neuropathic pain	CCI	Anti-hyperalgesic	[135]
		CCI-IoN	Anti-hyperalgesic (miR-223-3p)	[136]
miR-301	Neuropathic pain	SNI	Hyperalgesic	[137]
miR-320	Neuropathic pain	CCI	Hyperalgesic (miR-320a)	[138]
miR-330	Neuropathic pain	CCI	Hyperalgesic (miR-330-3p)	[139]
miR-365	Inflammatory pain	CFA	Hyperalgesic (miR-365-3p)	[140]
miR-382	Neuropathic pain	SCI	Hyperalgesic (miR-382-5p)	[141]
		CCI		[142]
miR-500	Neuropathic pain	CIPN (Paclitaxel)	Hyperalgesic	[143]
		L5-VRT		
miR-539	Neuropathic pain	CCI	Anti-hyperalgesic	[144]
miR-541	Neuropathic pain	bCCI	Hyperalgesic	[145]
miR-544	Inflammatory pain	AIA-CFA	Anti-hyperalgesic (miR-544-3p)	[146]
	Neuropathic pain	CCI	Hyperalgesic	[147]
miR-672	Neuropathic pain	BTZ	Hyperalgesic (miR-672-5p)	[148]

**Table 2:** List of siRNAs employed in the treatment of several chronic pain disorders. Abbreviations: CCI (chronic constriction injury), ERK (extracellular signal-regulated kinase), STAT3 (signal transducer and activator of transcription 3), CFA (complete Freund’s adjuvant), NF-κB (nuclear factor kappa-light-chain-enhancer of activated B cells), IL-1β (interleukin 1 beta), DPN (diabetic peripheral neuropathy), PARP1 (poly-ADP-ribose- polymerase 1), CIPN (chemotherapy-induced neuropathic pain), SNL (spinal nerve ligation), BCP (bone cancer pain), PDN (painful diabetic neuropathy), TLR4 (Toll-like receptor 4), PANX1 (pannexin 1), NLRP3 (NOD-like receptor family pyrin domain containing 3), SDH (spinal dorsal horn), IBA-1 (ionized calcium binding adapter molecule 1), PKC (protein kinase C), PI3K (phosphoinositide 3-kinase), PKB (protein kinase B), AAV (adeno-associated virus), SCI (spinal cord injury), LPS (lipopolysaccharide), CCL2 (C-C motif chemokine ligand 2), KCC2 (potassium chloride cotransporter 2), CACNA1H (calcium voltage-gated channel subunit alpha1 H), p-ERK (phosphorylated extracellular signal-regulated kinase), GFAP (glial fibrillary acidic protein), and OX42 (OX42 antigen/CD11b).

siRNA	Pain Condition	Pain Model	Effects on Pain	References
PKM2-siRNA	Neuropathic pain	CCI	Reduced mechanical sensitivity and thermal pain response associated with decreased ERK and STAT3 activation.	[169]
NR2B-siRNA	Inflammatory pain	CFA	Attenuating nociceptive responses.	[170]
NAMPT-siRNA	Inflammatory pain	CFA	Pain relief is achieved through the inhibition of the NF-κB/IL-1β inflammatory pathway.	[171]
PARP1-siRNA	Neuropathic pain	DPN	*PARP1* silencing reduces neuropathic symptoms.	[172]
IFT52-siRNA	Neuropathic pain	CIPN (Paclitaxel)	A decrease in primary cilia was correlated with an elevated mechanical nociceptive threshold.	[173]
IFT88-siRNA				
IKBKB-siRNA	Neuropathic pain	SNL	Mechanical allodynia was blocked, and the release of pro-inflammatory mediators driven by NF-κB was reduced.	[174]
TLR4-siRNA	Cancer pain	BCP	Intrathecal injection of TLR4-siRNA diminished nociception induced by Walker 256 cells.	[175]
PANX1-siRNA	Neuropathic pain	CCI	Knockdown of PANX1 in Schwann cells alleviated neuropathic pain.	[176]
TBK1-siRNA	Neuropathic pain	PDN	TBK1 activates the NF-κB pathway, triggers NLRP3 activation, causes microglia pyroptosis, all of which can be reversed by TBK1-siRNA injection.	[177]
Neurexin 2-siRNA	Inflammatory pain	CFA	Intrathecal neurexin 2-siRNA reduced CFA-induced mechanical and thermal hyperalgesia and decreased the expression of glutamate receptors in the SDH.	[178]
TRAF6-siRNA	Inflammatory pain	CFA	TRAF6-siRNA reduced CFA-induced allodynia and reversed the increase in IBA-1 expression.	[179]
NMUR2-siRNA	Cancer pain	BCP	NMUR2-siRNA alleviates BCP through the inactivation of the PKC/ERK and PI3K/PKB signaling pathways.	[180]
TRPV1-siRNA	Neuropathic pain	CIPN (Paclitaxel)	Intrathecal TRPV1-siRNA administration reduced paclitaxel-induced mechanical allodynia/hyperalgesia and thermal hyperalgesia.	[181]
	Cancer pain	BCP	Intrathecal administration of AAV-mediated TRPV1-siRNA enhanced both mechanical and thermal thresholds.	[182]
BRD4-siRNA	Neuropathic pain	CIPN (Vincristine)	The transfection of BRD-4-siRNA alleviated neuropathic pain caused by vincristine.	[183]
NR1-siRNA	Inflammatory pain	CFA	NR1-siRNA effectively reduced the nociceptive response induced by CFA stimulation.	[184]
STAT3-siRNA	Inflammatory pain	LPS	Blocking STAT3 activity reduced mechanical allodynia and was associated with fewer reactive astrocytes in the SDH.	[185]
TDAG8-siRNA	Cancer pain	BCP	Intrathecal siRNA-TDAG8 reduced BCP behaviors during both the onset and maintenance phases.	[186]
RAB11A-siRNA	Inflammatory pain	CFA	The injection of RAB11A-siRNA into the SDH led to a significant analgesic effect following CFA injection.	[187]
SHP1-siRNA	Inflammatory pain	CFA	SHP1-siRNA alleviated CFA-induced pain.	[188]
IL-36γ siRNA	Inflammatory pain	CFA	IL-36γ-siRNA significantly reduced chronic inflammatory pain behaviors induced by CFA.	[189]
CCL2-siRNA	Neuropathic pain	SCI	In vivo depletion of CCL2 reduced the intensity of chronic spinal compression and its associated pain.	[190]
KCC2-siRNA	Inflammatory pain	CFA	Intrathecal administration of KCC2-siRNA in naïve rats decreased KCC2 expression in the spinal cord, resulting in heightened pain behaviors and disrupted inhibitory synaptic transmission.	[191]
CXCR2-siRNA	Inflammatory pain	CFA	Perisciatic nerve injection of CXCR2-siRNA reduced CFA-induced mechanical allodynia and thermal hyperalgesia.	[192]
MT-I-siRNA	Inflammatory pain	CFA	Treatment with MT-I-siRNA prior to CFA injection or shortly after CCI significantly reduced mechanical allodynia and thermal hyperalgesia.	[193]
TRPM2-siRNA	Neuropathic pain	CCI	Treatment with TRPM2-siRNA during the early phase after CCI reduced injury-induced neuropathic pain.	[194]
ASIC3-siRNA	Inflammatory pain	CFA	ASIC3-siRNA exerts strong analgesic effects against primary inflammation-induced hyperalgesia.	[195]
CACNA1H-siRNA	Inflammatory pain	CFA	CACNA1H knockdown alleviated inflammatory pain.	[196]
CX3CR1-siRNA	Neuropathic pain	SNL	CX3CR1-siRNA treatment reduced microglial activation in the SDH, lowered pro-inflammatory mediators, and significantly decreased mechanical allodynia.	[197]
Vimentin-siRNA	Neuropathic pain	CCI	Vimentin knockdown reduced p-ERK upregulation, decreased vimentin expression, and lowered the release of pro-inflammatory cytokines.	[198]
PDGF-siRNA	Cancer pain	BCP	Intrathecal injection of PDGF-siRNA alleviated thermal and mechanical hyperalgesia in BCP rats.	[199]
PI3KCB-siRNA	Cancer pain	BCP	Silencing of PI3KCB using siRNA led to a reduction in the expression of GFAP and OX42.	[200]

**Table 3:** List of dysregulated lncRNAs across chronic pain disorders. Abbreviations: CFA (complete Freund’s adjuvant), lncRNA (long non-coding RNA), MEG3 (maternally expressed gene 3), TRPV1 (transient receptor potential vanilloid 1), SNL (spinal nerve ligation), DRG (dorsal root ganglion), NEAT1 (nuclear enriched abundant transcript 1), mRNA (messenger RNA), TNFAIP1 (tumor necrosis factor alpha induced protein 1), AKT (protein kinase B), CREB (cAMP response element binding protein), BCP (bone cancer pain), siRNA (small interfering RNA), miRNA (microRNA), CXCL13 (C-X-C motif chemokine ligand 13), SCI (spinal cord injury), CXCR5 (C-X-C chemokine receptor type 5), CCI (chronic constriction injury), WNT5A (Wnt family member 5A), JMJD1A (Jumonji domain containing 1A), bCCI (bilateral chronic constriction injury), SOCS3 (suppressor of cytokine signaling 3), JAK2 (Janus kinase 2), STAT3 (signal transducer and activator of transcription 3), SNL (spinal nerve ligation), SNHG1 (small nucleolar RNA host gene 1), pSNL (partial spinal nerve ligation), BAI1 (brain-specific angiogenesis inhibitor 1), EZH2 (enhancer of zeste homolog 2), CXCL9 (C-X-C motif chemokine ligand 9), and c-Fos (FBJ murine osteosarcoma viral oncogene homolog).

lncRNA	Pain Condition	Pain Model	Effects on Pain	References
lncRNA MEG3	Inflammatory pain	CFA	lncRNA MEG3 is negatively correlated with *TRPV1* mRNA in the DRG and SDH of CFA-induced rats. Therefore, the intrathecal delivery of a lentivirus overexpressing MEG3 significantly suppressed TRPV1 expression and relieved chronic inflammatory pain.	[238]
lncRNA NEAT1	Neuropathic pain	SNL	NEAT1 lncRNA regulated the expression of pro-inflammatory genes in the DRG of rats with neuropathic pain. NEAT1 increased the expression of pro-inflammatory genes by stabilizing its associated mRNAs in neuropathic pain.	[239]
lncRNA XIST	Inflammatory pain	CFA	The inhibition of the lncRNA XIST alleviated inflammatory pain by inhibiting satellite glial cell activation and inflammation.	[240]
lncRNA p21	Neuropathic pain	SNL	LncRNA p21 aggravated neuropathic pain by increasing TNFAIP1 expression and suppressing the AKT/CREB pathway.	[122]
lncRNA NONRATT014888.2	Cancer pain	BCP	lncRNA NONRATT014888.2 is highly expressed in tibia-related DRGs of BCP rats. Its downregulation by siRNA in BCP rats significantly reduced hind-paw mechanical pain hypersensitivity.	[241]
lncRNA NONRATT009773.2	Cancer pain	BCP	lncRNA NONRATT009773.2 was significantly up-regulated in BCP model. Depletion of lncRNA NONRATT009773.2 reduced BCP, while its overexpression triggered pain-like symptoms in naïve rats. Additionally, lncRNA NONRATT009773.2 acted as a miRNA sponge to absorb miR-708-5p and up-regulated the downstream target CXCL13, which plays a crucial role in hyperalgesia.	[242]
lncRNA PVT	Neuropathic pain	SCI	In the SCI model, PVT1 depletion significantly alleviated neuropathic pain, astrocytic activation, and reduced the expression of CXCL13/CXCR5.	[243]
lncRNA CRNDE	Neuropathic pain	CCI	lncRNA CRNDE intensified neuropathic pain in CCI rats by regulating the miR-146a-5p/WNT5A signaling pathway.	[244]
lncRNA FTX	Neuropathic pain	CCI	lncRNA FTX alleviated neuropathic pain by targeting miR-320a.	[138]
lncRNA PCAT19	Neuropathic pain	CCI	lncRNA PCAT19 influenced neuropathic pain by modulating the miR-182-5p/JMJD1A pathway.	[245]
lncRNA DILC	Neuropathic pain	bCCI	Suppression of lncRNA DILC alleviated neuropathic pain through the regulation of the *SOCS3*/JAK2/STAT3 pathway.	[246]
lncRNA SNHG1	Neuropathic pain	SNL	The inhibition of *SNHG1* reduced the progression of neuropathic pain, while its overexpression was sufficient to trigger neuropathic pain symptoms in naïve rats.	[247]
Lncenc1	Neuropathic pain	pSNL	Knockdown of Lncenc1 reduced the development and maintenance of mechanical and thermal hyperalgesia in pSNL mice, accompanied by increased BAI1 expression and decreased EZH2 expression in microglia.	[248]
lncRNA NONRATT021203.2	Cancer pain	BCP	lncRNA NONRATT021203.2 was increased in BCP rats and silencing it with siRNA reduced significantly hyperalgesia. lncRNA NONRATT021203.2 targeted CXCL9, which was also increased in BCP rats.	[249]
lncRNA 51325	Cancer pain	BCP	The overexpression of lncRNA 51325 significantly alleviated mechanical allodynia in BCP rats, while its knockdown induced pain behaviors and anxiety-like responses in naïve rats.	[250]
lncRNA 71132	Cancer pain	BCP	Spinal lncRNA 71132 was significantly upregulated in BCP. Its knockdown reversed BCP behaviors and reduced spinal c-Fos neuronal sensitization, while overexpression in naïve rats induced pain behaviors and heightened c-Fos sensitization. Additionally, lncRNA 71132 modulated BCP by inversely regulating miR-143-5p processing, with increased lncRNA 71132 expression leading to decreased miR-143 levels under BCP conditions.	[251]

**Table 4:** List of dysregulated circRNAs in chronic pain conditions. Abbreviations: CIVP (chronic inflammatory visceral pain), GFAP (glial fibrillary acidic protein), CFA (complete Freund’s adjuvant), DPN (diabetic peripheral neuropathy), DRG (dorsal root ganglion), STZ (streptozotocin), CCI (chronic constriction injury), NF-κB (nuclear factor kappa-light-chain-enhancer of activated B cells), IL-1β (interleukin 1 beta), IL-6 (interleukin 6), TNF-α (tumor necrosis factor alpha), SLICK (sequence like an intermediate conductance K channel), ENO1 (enolase 1), DHX9 (DEAH-box helicase 9), UBR5 (ubiquitin protein ligase E3 component N-recognin 5), ALB (albumin), COX-2 (cyclooxygenase 2), IL-10 (interleukin 10), CCI-IoN (chronic constriction injury of the infraorbital nerve), IST1 (increased sodium tolerance 1), LC3-II (microtubule-associated protein 1 light chain 3-II), p62 (Sequestosome 1-SQSTM1-), 3′-UTR (3′-untranslated region), KCNK1 (potassium channel, two-pore domain subfamily K, member 1), SNI (spared nerve injury), GAD65 (glutamate decarboxylase 65), NK1R (neurokinin 1 receptor), SNL (spinal nerve ligation), VEGFB (vascular endothelial growth factor B), Ybx1 (Y-Box binding protein 1), Wnt5a (Wnt family member 5A), LPAR3 (lysophosphatidic acid receptor 3), and BCP (bone cancer pain).

circRNA	Pain Condition	Pain Model	Effects on Pain	References
circRNA_02767	Inflammatory pain	CIVP	Moxibustion alleviated visceral pain in CIVP rats by modulating the circRNA_02767/rno-miR-483-3p/GFAP network in the spinal cord, thereby reducing central sensitization.	[286]
circRNA-Filip1l	Inflammatory pain	CFA	This study revealed that chronic inflammatory pain induced by CFA significantly upregulated circRNA-Filip1l expression in spinal neurons. Inhibiting this increase alleviated nociceptive behaviors, while its overexpression in naïve mice replicated pain responses, lowering thermal and mechanical nociceptive thresholds.	[290]
circHIPK3	Neuropathic pain	DPN	The research found that circHIPK3 is highly abundant in the serum of diabetes patients with neuropathic pain and in the DRG of STZ-induced diabetic rats. Silencing circHIPK3 alleviated neuropathic pain in diabetic rats by modulating neuroinflammation. circHIPK3 negatively regulated miR-124. Notably, inhibiting miR-124 reversed the pain relief and neuroinflammation reduction caused by circHIPK3 knockdown in diabetic rats.	[291]
circGRIN2B	Neuropathic pain	CCI	Overexpression of circGRIN2B has been shown to alleviate neuropathic pain by reducing mechanical and thermal hyperalgesia. This upregulation also significantly decreases pro-inflammatory cytokine levels (IL-1β, IL-6, and TNF-α) in the DRG. These findings suggest that circGRIN2B may mitigate neuropathic pain by modulating the NF-κB/SLICK pathway.	[292]
circSMEK1	Neuropathic pain	CCI	The results demonstrated that circSMEK1 and TXNIP were upregulated in neuropathic pain. Knockdown of circSMEK1 increased the claw retraction threshold and decreased claw retraction latency in rats. Additionally, circSMEK1 knockout reduced pro-inflammatory cytokines (e.g., TNF-α, IL-1β, and IL-6) in the spinal cord, suppressed microglial activation, and promoted microglial polarization toward the anti-inflammatory phenotype. Conversely, circSMEK1 upregulation had the opposite effects.	[293]
circZNF609	Neuropathic pain	CCI	This study demonstrated that circZNF609 exacerbates neuropathic pain progression by promoting the expression of pro-inflammatory factors through the miR-22-3p/ENO1 axis.	[294]
ciRNA-Fmn1	Neuropathic pain	CCI	The downregulation of ciRNA-Fmn1, resulting from altered DHX9 binding to DNA tandem repeats, contributed to the development of neuropathic pain by negatively regulating UBR5-mediated ALB expression in the spinal dorsal horn.	[295]
circ_0005075	Neuropathic pain	CCI	circ_0005075 was upregulated in CCI rat models. Knockdown of circ_0005075 attenuated neuropathic pain behaviors, including mechanical and thermal hyperalgesia. Furthermore, loss of circ_0005075 reduced neuroinflammation by targeting COX-2, IL-6, and TNF-α, while promoting the expression of IL-10.	[296]
circ_lrrc49	Neuropathic pain	CCI-IoN	Knockdown of circ_lrrc49 using siRNA decreased IST1 expression, elevated LC3-II and p62 levels, and increased the number of autophagosomes. This also induced orofacial mechanical hypersensitivity, an effect that could be reversed by IST1 overexpression.	[297]
ciRS-7	Neuropathic pain	CCI	ciRS-7 is associated with the progression of neuropathic pain, partly by upregulating autophagy and inflammation in CCI rats. Furthermore, ciRS-7 regulated neuropathic pain progression by sponging miR-135a-5p. In CCI rats, inhibition of miR-135a-5p reduced autophagy and pro-inflammatory cytokines, thereby alleviating neuropathic pain.	[113]
ciRNA-Kat6b	Neuropathic pain	CCI	Peripheral nerve injury downregulated ciRNA-Kat6b in the spinal horn of male mice. Restoring ciRNA-Kat6b expression alleviated CCI-induced pain hypersensitivities. The downregulation of ciRNA-Kat6b decreased the binding of miRNA-26a to ciRNA-Kat6b, while increasing its binding to the 3′-UTR of *KCNK1* mRNA, leading to the degradation of *KCNK1* mRNA and a reduction in Kcnk1 protein levels in the dorsal horn of neuropathic pain mice.	[298]
circFhit	Neuropathic pain	SNI	circFhit, an exon-intron circRNA in GABAergic neurons, reduced inhibitory transmission in the spinal dorsal horn, contributing to neuropathic pain after SNI. CircFhit downregulated GAD65 expression and induced hyperexcitation in NK1R^+^ neurons.	[299]
circAnks1a	Neuropathic pain	SNL	SNL upregulated circAnks1a in dorsal horn neurons, enhancing VEGFB expression through dual mechanisms. In the nucleus, circAnks1a binds the VEGFB promoter, promoting Ybx1 recruitment and transcription. In the cytoplasm, it acts as a miR-324-3p sponge, preventing VEGFB downregulation. Elevated VEGFB protein enhances neuronal excitability, contributing to nerve injury-induced pain.	[300]
circRNA cZRANB1	Neuropathic pain	CCI	cZRANB1 promoted neuropathic pain in CCI rat models by modulating Wnt5a/β-Catenin signaling via the miR-24-3p/LPAR3 axis.	[301]
circSlc7a11	Cancer pain	BCP	The circRNA circSlc7a11 regulated BCP progression in rats by modulating Walker-256 cell proliferation and apoptosis through multiple signaling pathways.	[302]

**Table 5:** Comparative overview of ncRNA types involved in chronic pain: stability, specificity, and translational challenges. Abbreviations: ncRNA (non-coding RNA), miRNA (microRNA), siRNA (small interfering RNA), circRNA (circular RNA), and lncRNA (long non-coding RNA).

ncRNA Class	Stability	Specificity	Delivery Challenges	Clinical Translation Strength	Limitations	References
miRNA	Moderate	High	Moderate (requires carriers)	Abundant data in pain models Good biomarker potential	Off-target effects (redundancy in miRNA-mRNA interactions)	[303]
siRNA	High	Very high	Moderate to high (delivery to neurons remains challenging)	Potent gene silencing	Immune activation Transient effects	[304]
circRNA	High	Moderate	Moderate (emerging delivery methods)	Excellent stability Novel biomarker potential	Function not understood Complex to manipulate	[305]
lncRNA	Low	High	High (large size and poor uptake limit therapeutic delivery)	Highly specific functions in neuronal and immune pathways	Limited in vivo data Complex structure- function relationships	[306]

## Data Availability

Not applicable. No new data were generated.

## Abbreviations

The following abbreviations are used in this manuscript:

3′-UTR	3′-untranslated region
AAV	Adeno-associated virus
AGO	Argonaute
AGO2	Argonaute 2
AIA	Adjuvant-induced arthritis
AKT	Protein kinase B
ALB	Albumin
AP-1	Activator protein 1
ASIC	Acid-sensing ion channel
BAI1	Brain-specific angiogenesis inhibitor 1
bCCI	Bilateral chronic constriction injury
BCP	Bone cancer pain
BTZ	Bortezomib
CACNA1H	Calcium voltage-gated channel subunit alpha1 H
CCD	Chronic compression of the DRG
CCI	Chronic constriction injury
CCI-IoN	Chronic constriction injury of the infraorbital nerve
CCL2	C-C motif chemokine ligand 2
ceRNA	Competing endogenous RNA
CFA	Complete Freund’s adjuvant
c-Fos	FBJ murine osteosarcoma viral oncogene homolog
ChIRP	Chromatin isolation by RNA purification
ciRNA	Circular intronic RNA
circRNA	Circular RNA
CIPN	Chemotherapy-induced neuropathic pain
CIVP	Chronic inflammatory visceral pain
CLIP	Class II-associated invariant chain peptide
CNS	Central nervous system
COX-2	Cyclooxygenase 2
CRC	Colorectal cancer
CREB	cAMP response element binding protein
CXCL13	C-X-C motif chemokine ligand 13
CXCL9	C-X-C motif chemokine ligand 9
CXCR5	C-X-C chemokine receptor type 5
DGCR8	DiGeorge Syndrome critical region 8
DNA	Deoxyribonucleic acid
DPN	Diabetic peripheral neuropathy
DRG	Dorsal root ganglion
DHX9	DEAH-box helicase 9
dsRNA	Double-strand RNA
ecircRNA	Exonic circRNA
EIciRNA	Exon-intron circRNA
ENO1	Enolase 1
ERK	Extracellular signal-regulated kinase
EZH2	Enhancer of zeste homolog 2
FUS	Fused in sarcoma
GAD65	Glutamate decarboxylase 65
GFAP	Glial fibrillary acidic protein
GTP	Guanosine triphosphate
IASP	International Association for the Study of Pain
IBA-1	Ionized calcium binding adapter molecule 1
IFT52	Intraflagellar transport 52
IFT88	Intraflagellar transport 88
IKBKB	Inhibitor of NF-κB
IL-1β	Interleukin 1 beta
IL-10	Interleukin 10
IL-6	Interleukin 6
IRES	Internal ribosome entry site
IST1	Increased sodium tolerance 1
JAK2	Janus kinase 2
JMJD1A	Jumonji domain containing 1A
KCC2	Potassium chloride cotransporter 2
KCNK1	Potassium channel, two-pore domain subfamily K, member 1
L5-VRT	L5 ventral root transection
lncRNA	Long non-coding RNA
LC3-II	Microtubule-associated protein 1 light chain 3-II
LPAR3	Lysophosphatidic acid receptor 3
LPS	Lipopolysaccharide
LTD	Long-term depression
LTP	Long-term potentiation
m6A	N6-methyladenosine
MBL	Mannose-binding lectin
MEG3	Maternally expressed gene 3
miRNA	MicroRNA
mRNA	Messenger RNA
NAMPT	Nicotinamide phosphoribosyltransferase
ncRNA	Non-coding RNA
NEAT1	Nuclear enriched abundant transcript 1
NF-κB	Nuclear factor kappa-light-chain-enhancer of activated B cells
NK1R	Neurokinin 1 receptor
NLRP3	NOD-like receptor family pyrin domain containing 3
NR2B	N-methyl-D-aspartate receptor subunit 2B
ORF	Open reading frame
OX42	OX42 antigen/CD11b
p62	Sequestosome 1 (SQSTM1)
PANX1	Pannexin 1
PARP1	Poly(ADP-ribose) polymerase 1
p-ERK	Phosphorylated extracellular signal-regulated kinase
PDN	Painful diabetic neuropathy
PI3K	Phosphoinositide 3-kinase
PI3KCB	Phosphoinositide 3-kinase catalytic subunit beta
PKB	Protein kinase B
PKC	Protein kinase C
PKM2	Pyruvate kinase M2
PNS	Peripheral nervous system
PRC2	Polycomb repressive complex 2
pre-mRNA	Precursor messenger RNA
pre-miRNA	Precursor microRNA
pri-miRNA	Primary microRNA
pSNL	Partial spinal nerve ligation
PVT1	Plasmacytoma variant translocation 1
Ran	Ras-related nuclear protein
RBP	RNA-binding protein
RdRPs	RNA-dependent RNA polymerase
RISC	RNA-induced silencing complex
RNA	Ribonucleic acid
RNA Pol II	RNA polymerase II
RNA Pol III	RNA polymerase III
RNA-seq	RNA sequencing
SCI	Spinal cord injury
SDH	Spinal dorsal horn
SGK3	Serum/glucocorticoid regulated kinase family member 3
siRNA	Small interfering RNA
SLICK	Sequence like an intermediate conductance K channel
SNHG1	Small nucleolar RNA host gene 1
SNI	Spared nerve injury
SNL	Spinal nerve ligation
SOCS3	Suppressor of cytokine signaling 3
STAT3	Signal transducer and activator of transcription 3
STZ	Streptozotocin
TET1	Ten-eleven translocation methylcytosine dioxygenase 1
TLR4	Toll-like receptor 4
TNFAIP1	Tumor necrosis factor alpha induced protein 1
TNF-α	Tumor necrosis factor alpha
TRBP	Transactivation response element RNA-binding protein
TRPV1	Transient receptor potential vanilloid 1
U1-snNRP	U1 small nuclear ribonucleoprotein
UBR5	Ubiquitin protein ligase E3 component N-recognin 5
VEGFB	Vascular endothelial growth factor B
WNT5A	Wnt family member 5A
Ybx1	Y-Box binding protein 1
XPO5	Exportin 5
